# Direct air capture of CO_2_: A response to meet the global climate targets

**DOI:** 10.1557/s43581-021-00005-9

**Published:** 2021-06-05

**Authors:** Mihrimah Ozkan

**Affiliations:** 1 Department of Electrical and Computer Engineering, University of California Riverside, Riverside, CA 92521 USA; 2 Department of Chemistry, University of California Riverside, Riverside, CA 92521 USA; 3 Materials Science and Engineering Program, University of California Riverside, Riverside, CA 92521 USA

**Keywords:** carbon dioxide, absorbent, absorption, environment, storage, renewable

## Abstract

**Highlights:**

DAC can help deal with difficult to avoid emissions. Large-scale deployment of DAC requires serious government, private, and corporate support and investment particularly to offset the capital cost as well as operational costs. Further optimizations to the costs can be found in choice of energy source as well as advances in CO_2_ capture technology such as high capacity and selectivity materials, faster reaction kinetics, and ease of reusability.

**Abstract:**

Direct air capture (DAC) technologies are receiving increasing attention from the scientific community, commercial enterprises, policymakers and governments. While deep decarbonization of all sectors is required to meet the Paris Agreement target, DAC can help deal with difficult to avoid emissions (aviation, ocean-shipping, iron-steel, cement, mining, plastics, fertilizers, pulp and paper). While large-scale deployment of DAC discussions continues, a closer look to the capital and operational costs, different capture technologies, the choice of energy source, land and water requirements, and other environmental impacts of DAC are reviewed and examined. Cost per ton of CO_2_ captured discussions of leading industrial DAC developers with their carbon capture technologies are presented, and their detailed cost comparisons are evaluated based on the choice of energy operation together with process energy requirements. Validation of two active plants’ net negative emission contributions after reducing their own carbon footprint is presented. Future directions and recommendations to lower the current capital and operational costs of DAC are given. In view of large-scale deployment of DAC, and the considerations of high capital costs, private investments, government initiatives, net zero commitments of corporations, and support from the oil companies combined will help increase carbon capture capacity by building more DAC plants worldwide.

**Graphic abstract:**

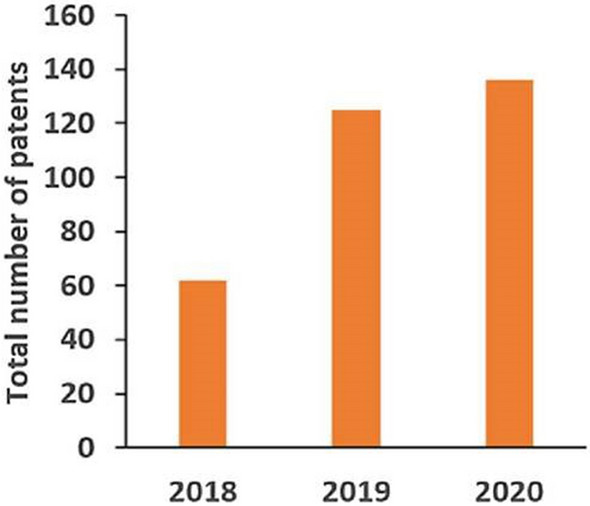

## Discussion

- DAC technologies are not profitable yet. Can they be profitable at all?
- How can capital costs for DAC plants be reduced?
- How can the operational costs of DAC be lowered and which advancements are needed in system design, carbon capture materials, and capture and release processes to help with cost reduction?
- What are some of the short term and long-term steps moving forward with large-scale deployment of DAC?
- Should we be alerted about the recent interest from the oil companies? While helping to pump more oil with CO_2_ can we still meet the net zero emission requirements?
- Do we know enough about the carbon capture system life cycle for full-life-cycle assessments of existing plants?
- Which parts of the DAC process are energy or cost demanding? Is it really the capture component, storage, transportation, or sequestration?
- What is the techno-economic impact of the DAC plant location choice?
- What are the immediate and long-term steps needed from investors, corporates, policy makers and governments?

## Commentary

**Figure 1:**
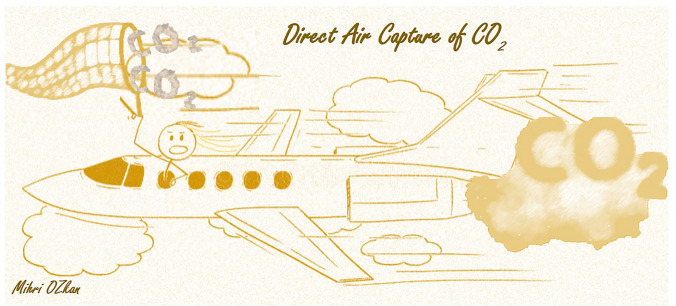
Cartoon expression for DAC.

During the Covid-19 lockdowns, annual global fossil CO_2_-emissions declined from 2019 to 2020 by 7%.^1^ Even though this decline is a momentary effect, it also demonstrates possible control of the CO_2_ emission, if it is wanted. Today, according to the Mauna Loa Observatory in Hawaii, the atmospheric CO_2_ levels increased to an average of nearly 420 parts per million, about 50% higher than before the Industrial Revolution levels (280 ppm). In December 2015, talks at the Paris meeting have identified the need for immediate action aimed at reducing CO_2_ emissions to limit the increase of global temperatures between 1.5 and 2 °C.^2^ According to Berkeley Earth and UK Hadley Center, the global mean temperature in 2020 is estimated as 1.27 °C above the average temperature in the late nineteenth century. In order to meet below 2 °C climate goals, there needs to be nearly 10 GtCO_2_ removed globally per year until 2050, and after 2050 nearly 20 GtCO_2_ removed globally per year until 2100.^2, 3^ Therefore, meeting climate goals demand fast decarbonization act and rapid deployment of “negative carbon technologies” such as technologies that can remove CO_2_ from the atmosphere -direct air capture (DAC, Fig. 1).

## Commercial status of DAC

There are three leading industrial DAC developers, today: (1) Climeworks in Switzerland, (2) Carbon Engineering in Canada, and (3) Global Thermostat in USA. Carbon Engineering uses a liquid solvent system for CO_2_ capture while Climeworks and Global Thermostat uses a solid sorbent system for CO_2_ capture. With solvent DAC technology (Carbon Engineering), air first enters from the inlets and passes through the contactors. Contactors are made from packed PVC sheets which are wetted with liquid solvent that is gravity fed from the top of the system. PVC sheets are packed in a large surface area arrangement which allows for more liquid–air contact to optimize CO_2_ capture. Air containing CO_2_ flows through these surfaces and then CO_2_ molecules encounter the liquid and convert to carbonate. The next step is recovering CO_2_ from the carbonate salt. While KOH solvent is circulated back to the contactors, carbonate pellets are dried inside the steam slaker at nearly 300 °C, and then feed into the calciner unit at 900 °C. After recovery, CO_2_ gas is collected inside the condenser and later compressed into the storage. This heat intense calcination process draws the most energy. It has been reported that for 15 MPa CO_2_ output, system requires either 8.81 GJ of natural gas, or 5.25 GJ of gas, and 366 kWh of electricity per ton of CO_2_ captured. Carbon Engineering capture technology cost range is estimated as $94–232/tCO_2_, and if the financial and gas price assumptions of (the capital recovery factor = 12% and $6/GJ) used, then technology costs would be $107–249/tCO_2_.^4^

Other leading industrial developer, Climeworks technology is based on a cyclic adsorption desorption process achieved using a porous high surface area material. During the first step, air fans draw air through the capture unit, and the CO_2_ in the air is bound at the surface of the filter material. CO_2_ free air leaves the filter. This is repeated until the filter is filled with CO_2_. The filter with the trapped CO_2_ is heated up to 100 °C and CO_2_ is released again. Released CO_2_ is concentrated at 99.9% purity from the filter. Energy requirement for this DAC process are around 2000 kWh/tCO_2_, or 400 kWh electrical and 1,600 kWh thermal. A $100/tCO_2_ is estimated to be reached within a decade.^5^

Jumping to the carbon capture wagon, based in Dublin, Silicon Kingdom Holdings together with Arizona State University plans to build 1,200 mechanical trees each with expected carbon capture capacity of 2.5 tCO_2_/year. 30 feet tall mechanical trees capture CO_2_ from the air as the wind passes through them. A group of 12 mechanical trees can collect 1 tCO_2_ daily. A pilot site planned in California can remove up to 36,500 tCO_2_ annually. A pilot site of mechanical trees is nearly equivalent to 1,825 households’ annual emissions, assuming average American household emits about 20 tCO_2_ annually.

## Can we reach to climate goals with DAC?

The deep decarbonization of industry is a necessity to become carbon neutral. Can DAC help to this effort as a potential negative emission technology? For a 1.5  °C goal, the cumulative negative emissions needed are between 450 and 1000 GtCO_2_ by 2100.^3^ In order to remove 1000 GtCO_2_ by 2100 (79 years from today), we now need nearly 13,000 Carbon Engineering plant partnered with Oxy Low Carbon Ventures in Permian Basin in Texas. This plant’s CO_2_ removal capacity per year is 1MtCO_2_ and it is expected to be fully operational by 2024. Using Climeworks joint partnership with Carbfix in Hellisheidi Iceland plant, nearly 4,000 tCO_2_ can be removed per year. In order to remove 1000 GtCO_2_ by 2100, today, nearly 3 million similar plants are needed. Estimated annualized capital costs, for a generic solid sorbent and liquid solvent DAC systems with a capacity of 1MtCO_2_ per year and with an estimated 30-year plant life, are projected at nearly $133 million and $126 million, respectively.^3^ To meet the global goals using DAC alone (remove 1000 GtCO_2_ by 2100), nearly 13,000 DAC plants with 1MtCO_2_ per year capacity are needed today, and thus earth needs nearly $1.7 Trillion (10^12^) (or $1.6 Trillion) capital investment. For the estimated cost analysis, operational costs are not included. Capital costs for equipment and commercialization costs are main considerations for DAC plants, especially during the scale-up stage. For the liquid solvent-based systems, the majority of the capital expenses are contactor arrays, oxy-fired calciner, slaker, causticizer, clarifier and condenser units. For the solid sorbent-based system, about 80% of the capital is associated with the nitrogen functionalized porous materials, and the remaining is associated with the oxy-fired calciner, vacuum pump and heat exchanger. From the operational costs perspectives, liquid solvent-based systems costs are slightly higher than the solid sorbent-based systems. This is mainly due to the high energy demands at the regeneration process along with the electricity required to run the fans. The capital and operational costs can be lowered for DAC systems with an improved system design as well as advances in solvent or sorbent materials with higher capture capacity, selectivity, and recyclability.

## Energy needs of DAC and cost analysis

A closer look needs to be given to the energy needs for DAC as air capture deals with an extremely low CO_2_ concentration (420 ppm) which is roughly 350 times lower than that found in a typical coal-based flue gas (12%). DAC process uses a more dilute stream, and it requires more work to separate the CO_2_ out of that more dilute stream than the more concentrated streams such as flue gas. Hence about three times more energy is required in terms of just the minimum work to capture CO_2_ from the atmosphere compared to the exhaust stream of the power plant. In order to capture comparable amounts of CO_2_ from the air nearly 300 times more contactor area is required for the DAC plant than from a power plant exhaust. Higher energy demands of the DAC plant adds to the operating costs which strongly correlates with the energy it takes to do the CO_2_ separation. Furthermore, contactors are a significant capital costs of the DAC plant, so it is always going to be more expensive to capture CO_2_ from the air compared to a flue gas source. Furthermore, CO_2_ selectivity of liquid solvent or solid sorbent could play an important role in a nitrogen and water vapor rich ambient air. This can lead to captured CO_2_ with varying purity percentage. For ease of transportation of CO_2_, it needs to be converted into a condensed phase which requires a gas compression step. CO_2_ in high purity is more compressible than low purity CO_2_. Depending on the type and efficiency of capture process, a purification step may be added and this also increases energy needs of the DAC operation.

A recent DAC cost analysis reports that^6^ for a solvent-based 1 MtCO_2_/year DAC system requires about 300 MW power where 80% is thermal and 20% is electric. In the cost calculations, only the capture, heat and power generation, and CO_2_ compression steps’ power requirements are considered. In order for DAC to be a true net negative emission technology that removes CO_2_ from the environment, the system needs to be powered with electricity from the renewable energy sources. In this report, capture costs based on different energy source operations are given as: $360-$620/tCO_2_ for nuclear energy; $360-$570/tCO_2_ for wind; $250-$440/tCO_2_ for geothermal energy; $430-$690/tCO_2_ for solar energy; and $300-$490/tCO_2_ for hybrid system with natural gas powered electric calciner. Based on all cost evaluations, geothermal powered DAC system has the lowest cost and in all calculations the cost of transportation and sequestration are neglected. All reports conclude that renewable energy source to power DAC is a key factor to lower the overall cost of DAC per tCO_2_. A caution with the various costs analyses are needed because of a very specific boundary conditions and assumptions have been used for a specific type of DAC systems.

## Funding for DAC

Private investor and company investments can help to scale up existing capture capacity of plants. Shopify invests annually $5 million in sustainability fund for the Carbon Engineering -backed by Bill Gates and Canadian oilsands investor Murray Edwards, will explore options for permanently storing CO_2_. Carbon Engineering also established partnerships with the Virgin Group and plan to deploy a plant in United Kingdom. Through more private investors such as BHP, First Round Capital, Starlight Ventures, Oxy Low Carbon Ventures, Chevron Technology Ventures, and Lowercase Capital, Carbon Engineering has secured nearly $91 million funding.

Climeworks using amine functionalized filter-based capture is selling CO_2_ removal services to corporate customers including Microsoft, Audi, Shopify and Stripe. Further Climeworks supplies aviation fuel for the Rotterdam The Hague airport. CO_2_ is also sold to customers such as Coca Cola. About $100 million private investor funding can also help for future expansions. Government support through German and Swiss ministries, EU Horizon 2020 program, and EU incubators programs are additional support for Climeworks. Additionally, several governments have approved several initiatives and funding programs to aid in the adoption of DAC and to solve current grand challenges. The USA recently announced its funding of $24 million to be used for R&D of DAC along with a newly reformed 45Q tax credit program. United Kingdom similarly announced their funding of 70 million euros for research in DAC and greenhouse gas removal while the European Union's funding of DAC concluded with their project "Store & Go". Lastly, the Canadian government has also demonstrated their support of DAC by donating $24 million to Carbon Engineering. In 2021, in Europe, emission trading cost is reported to raise to a record high of nearly $49/tCO_2_ that can financially benefit the DAC companies even more.

## Validation of DAC as a net negative emission technology

A closer look needs to be given to the net CO_2_ removed with the DAC systems over its full life cycle. A recent life-cycle assessment of DAC^7^ reports that Climeworks plants in Hinwil and Hellisheidi operate with carbon capture efficiencies of 85.4% and 93.1%, respectively. Both plants are, indeed, carbon negative. The main conclusion from this report is that chosen energy source to power the DAC plan will dictate the net CO_2_ removed from the air. Majority of the emissions are concluded as due to energy and nearly 8% is from plant and nearly 34% is from the adsorbents. Access to low carbon or renewable energy sources need to be a decision factor in choosing the location of the next DAC plant.

## Land and water requirements of DAC

Land and water consumptions are other considerations for DAC. For a modern liquid solvent DAC technology to capture 1 ton of CO_2_, the system uses nearly 1–7 tons of water. The water footprint of DAC is an important parameter together with required land size for a DAC plant. DAC plant does not require arable land and would not take away from food and farm production which allows for flexibility of location. However, the energy resource powering and water requirements may limit preferable location choices.^8^ Seasonal variability of temperature, humidity, wind, and air pollution content can affect the choice of capture method and land selection, as well. Carbon Engineering plant with Oxy Low, powered geothermally and with 1 MtCO_2_/year capacity, requires land area between 0.2–0.6 square kilometers.^9^ To remove 1000 GtCO_2_ by 2100, today, nearly 13,000 of these plants are needed. The total land area required in average is nearly 6.6 times of the size of New York City. Capturing a similar amount of CO_2_ by 2100 from forests (according to the World Resources Institute, forests absorb nearly 7.6 GtCO_2_/year and the world has nearly 4 billion hectares of forests), a land size of nearly 6.77 times of the size of the United States is required. Besides large land area requirements, forests will also need much more water sources compared to DAC, in addition to the time of development of trees.

## Other environmental impacts of DAC

Environmental impact of DAC, other than land and water, are reported based on material needs for plant construction (i.e. concrete, steel, stainless steel, aluminum, copper, plastics, insulation, paints, adsorbent) and energy footprint (energy to run DAC and energy for storage) to capture 1% of the annual global CO_2_ emissions.^7^ Adsorbents (4.8–84%) and energy footprint (0–92%, wind power considered as a best case) have the most environmental impact among all other considerations.^7^ Energy has the largest environmental impact-print with considerations of human toxicity, cancer, non-cancer; resource depletion and energy; mineral and metal; acidification, terrestrial and freshwater; particulate matter. Adsorbents have the second most environmental impact with considerations of resource depletion, energy; human toxicity, cancer, non-cancer; acidification, terrestrial and freshwater; eutrophication, freshwater, terrestrial; marine; photochemical ozone formation; and water scarcity. These predictions are important to consider for large-scale deployment of DAC.

## Sorbent technologies

**Figure 2:**
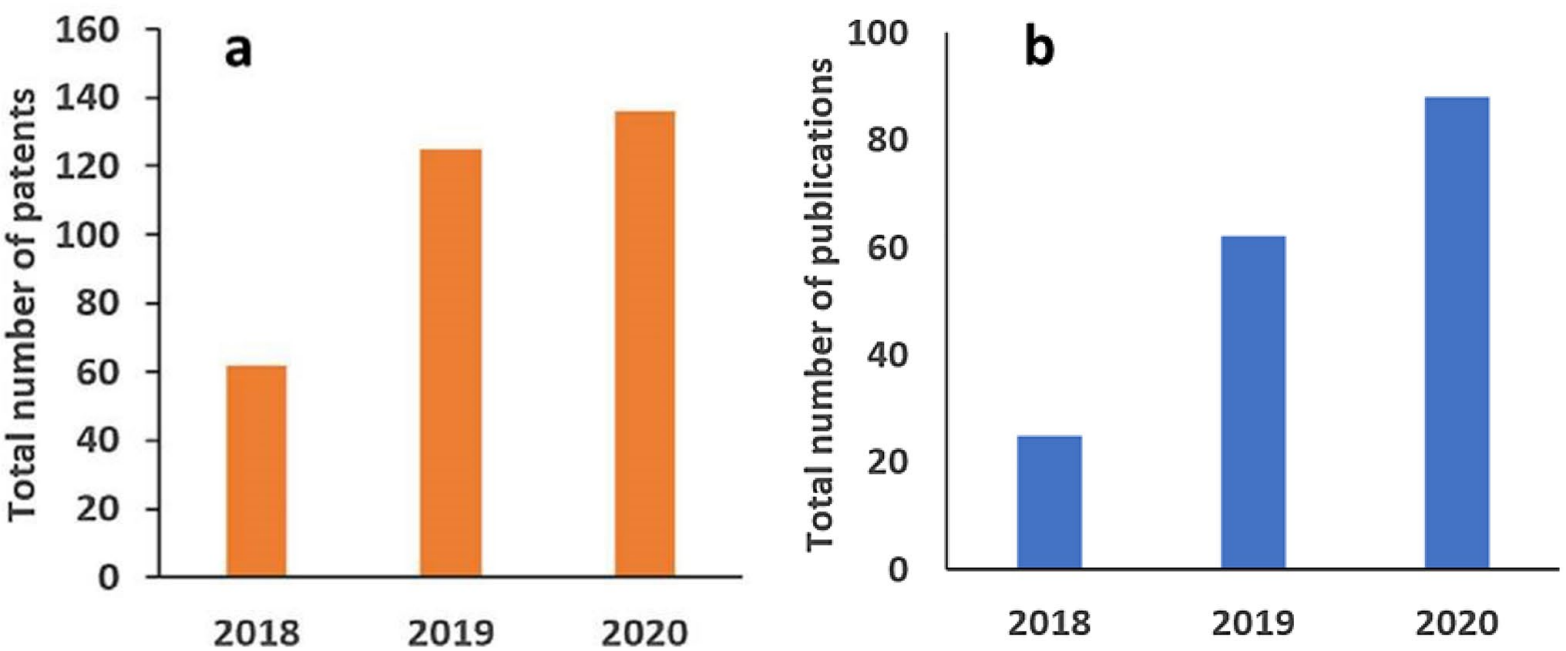
(a) Cumulative number of patents and patent applications and (b) Total number of publications on Direct Air Capture since 2018. The numbers listed here are obtained from Web of Science for the publications and Google Scholar for the patents and applications. (Generated by Mihrimah (Mihri) Ozkan).

Starting from a historical development of sorbent-based DAC concept introduced by Lackner in 1999^10^ up until today’s large-scale applied DAC technologies worldwide, it needs to be evaluated comprehensively to answer the standing question: How much can the DAC technology implementation help to meet the climate goals? Fig. 2 shows the rapid increase in the number of published papers and patents within the last three years in the field of DAC. Recently, a rapid growth of reports using groups of chemisorbents for CO_2_ capture from dilute gas streams such as ambient air, liquid and solid sorbents prepared from alkali and alkaline earth metal oxides and hydroxides, sorbents prepared from amines, and designed metal − organic frameworks (MOFs) have been reported.^11–14^ It has been proposed that physiosorbent materials such as zeolites, activated carbons, or MOFs typically perform poorly at low CO_2_ partial pressures, offering very small CO_2_ capture and low CO_2_ selectivity.^5^ As air capture deals with an extremely low CO_2_ concentration (420 ppm), roughly 350 times lower than that found in a typical coal-based flue gas, liquid solvent materials have proven to be much more effective for DAC processes so far. On the other hand, solid sorbent materials demand lower energy value compared to liquid solvent materials due to less required energy during the regeneration process based on the weaker-bonding nature of adsorption process. Among the solid sorbent materials, modified graphene-based sorbent materials have shown nearly 10 times more efficient CO_2_ adsorption compared to other active carbon, zeolites or MOFs.^11–14^ This is attributed to CO_2_ adsorption on both sides of each graphene sheet that provides potentially larger surface area for adsorption, and additionally with surface modification of graphene using nitrogen rich compounds can enhance the CO_2_ capture further. More recently, membrane separation is proposed and considered the most energy-efficient technique for CO_2_ separation among the various separation technologies. Moreover, membrane approach does not require special chemicals or sorbents. The utmost advantage is that membrane separation systems are scalable and can be a great tool for large-scale deployment of DAC.^13^

While developing better sorbent materials, it is essential to test these new sorbent materials at the natural CO_2_ partial pressures close to the atmospheric values or close to the pressures at the considered emitting source sites. Majority of the scientific literatures report CO_2_ capture tests mostly done with pure CO_2_ sources at high gas partial pressure values. Furthermore, depending on the location of application of carbon capture process, temperature plays an important role. Hence, at elevated temperatures, the loss of captured CO_2_ is very likely especially for solid sorbent materials. This can lead to the loss of CO_2_ back to the environment. The direct capture of CO_2_ from the air is possible, but technically and economically still challenging, primarily due to inherent difficulty of carbon capture at the extremely dilute concentration of atmospheric CO_2_ (420 ppm).

## Interest from oil companies

Emerging markets for captured CO_2_ are construction (carbon-rich aggregates for concrete) and fuels (conversion of captured CO_2_ to hydrocarbons). Hence, carbon capture has been garnering interest among oil companies. Global Thermostat jointly with ExxonMobil expects to remove 1 GtCO_2_/year using their amine modified monolith-based technology and reports planned additional scale up to remove 40 GtCO_2_/year. The French institute IFP Energies Nouvelles and the oil company Total invested nearly $40 million to improve the energy efficiency of carbon capture process. Oil companies Chevron and Equinor invested in Clean Carbon Solutions which claims that their system is 40% cheaper to operate and 20% cheaper to build than others’ systems based on monoethanolamine. Occidental targets to build the world’s largest DAC plant together with Carbon Engineering to capture nearly 1 MtCO_2_/year mainly to push crude-oil out of wells. According to Environmental Protection Agency, this amount is nearly equivalent to emission from more than 200,000 cars a year. A plant at this size is eligible for the 45Q tax credit (2018) and potentially eligible for the California low carbon fuel credit as well. In 2019, added total credits reached nearly to $180/tCO_2_, according to the International Energy Agency. An Italian oil group, Synhelion and ENI, team up to build their first commercial plant by 2025. This incredible attention and investment from oil companies towards the carbon capture technologies and the use of captured CO_2_ to expand oil production is also somewhat troubling and debatable, because it will potentially lead the way to produce more fossil fuels and emit more greenhouse gases.

## Final thoughts

In summary, based on these reported cost values, the DAC technologies do not seem to be profitable yet. Additionally, relying only on DAC technologies could result in a scapegoat, since policy-makers could use DAC technologies as a defense to postpone other high potential climate mitigation measures such as deep decarbonization of industry. Hence, it is of great importance to evaluate the environmental and economic performance of DAC technologies as accurately as possible at the commercial scale before large-scale global implementations begin to spread and other mitigation technologies are avoided. Another argument for using DAC is that it needs to use renewables to power for removing CO_2_ from the air, and thus they can reduce CO_2_ without experiencing an added air pollution cost. To lower the cost of DAC, low-cost solid sorbents and liquid solvents synthesis development at industry levels is necessary. Increased capture capacity, fast reaction kinetics, high selectivity for CO_2_, and stability and long lifetime of these materials can lower both capital and operational costs. Other ways to optimize costs is utilizing waste heat from other processes, shallow contactor design to minimize pressure drop, alternative regeneration methods including steam, microwave and vacuum-swing regeneration. After application of all advancements, it is still open for a debate that whether reforestation, reducing biomass burning, or reducing halogen, nitrous oxide, and methane emissions are a more cost-effective approach of mitigating climate change compared to the DAC of CO_2_.

Finally, in 2018, a new US federal tax credit—45Q, provides incentives for capturing and storing or reselling CO_2_. Furthermore, Microsoft’s announcement of $1 Billion investment to carbon capture and removal technologies, and Elon Musk’s $100 Million prize offer to the best carbon capture technology would certainly stimulate the activities in the DAC of CO_2_. In the best scenario, by capturing CO_2_ from the atmosphere economically and by making profit via selling captured CO_2_ to make fuel, plastics, carbon fiber, concrete, drinks and more, can this address the lingering question: Can the DAC of CO_2_ provide a free get-out-of-jail card for climate change and thus, help to mitigate global warming? Furthermore, since last year doubled increase in the number of large companies including; Amazon, Microsoft, Google, Apple, Facebook, Ford, BP, Shell, United, Nestle and more joining with “Net Zero” commitments is highly pleasing, yet, is it enough, and can all this help with the deep decarbonization efforts for all sectors?
